# The *Vibrio cholerae* Cytolysin Promotes Chloride Secretion from Intact Human Intestinal Mucosa

**DOI:** 10.1371/journal.pone.0005074

**Published:** 2009-03-31

**Authors:** Lucantonio Debellis, Anna Diana, Diletta Arcidiacono, Romina Fiorotto, Piero Portincasa, Donato Francesco Altomare, Carlo Spirlì, Marina de Bernard

**Affiliations:** 1 Department of General and Environmental Physiology, University of Bari, Bari, Italy; 2 Venetian Institute of Molecular Medicine, Padua, Italy; 3 Department of Internal Medicine, Section of Digestive Diseases, Yale University, New Haven, Connecticut, United States of America; 4 Clinica Medica “A. Murri”, Department of Internal and Public Medicine, University Medical School, Bari, Italy; 5 Department of Emergency and Organ Transplantation, General Surgery and Liver Transplantation Units, University of Bari, Policlinico, Bari, Italy; 6 Department of Biology, University of Padua, Padua, Italy; Charité-Universitätsmedizin Berlin, Germany

## Abstract

**Background:**

The pathogenicity of the *Vibrio cholerae* strains belonging to serogroup O1 and O139 is due to the production of virulence factors such as cholera toxin (CT) and the toxin-coregulated pilus (TCP). The remaining serogroups, which mostly lack CT and TCP, are more frequently isolated from aquatic environmental sources than from clinical samples; nevertheless, these strains have been reported to cause human disease, such as sporadic outbreaks of watery diarrhoea and inflammatory enterocolitis. This evidence suggested the possibility that other virulence factor(s) than cholera toxin might be crucial in the pathogenesis of *Vibrio cholerae*-induced diarrhoea, but their nature remains unknown. VCC, the hemolysin produced by virtually all *Vibrio cholerae* strains, has been proposed as a possible candidate, though a clear-cut demonstration attesting VCC as crucial in the pathogenesis of *Vibrio cholerae*-induced diarrhoea is still lacking.

**Methodology/Principal Findings:**

Electrophysiological parameters and paracellular permeability of stripped human healthy colon tissues, obtained at subtotal colectomy, mounted in Ussing chamber were studied in the presence or absence of VCC purified from culture supernatants of *V. cholerae* O1 El Tor strain. Short circuit current (I_SC_) and transepithelial resistance (R_T_) were measured by a computerized voltage clamp system. The exposure of sigmoid colon specimens to 1 nM VCC resulted in an increase of I_SC_ by 20.7%, with respect to the basal values, while R_T_ was reduced by 12.3%. Moreover, increase in I_SC_ was abolished by bilateral Cl^−^ reduction.

**Conclusion/Significance:**

Our results demonstrate that VCC, by forming anion channels on the apical membrane of enterocytes, triggers an outward transcellular flux of chloride. Such an ion movement, associated with the outward movement of Na^+^ and water, might be responsible for the diarrhoea caused by the non-toxigenic strains of *Vibrio cholerae*.

## Introduction

Cholera is an acute diarrhoeal infection caused mostly by ingestion of *Vibrio cholerae* O1 and O139 and it represents one of the most outbreak-prone diseases that continue to strike fear in the population wherever it occurs. Although the advent of oral rehydration therapy (ORT) has substantially improved the cholera case fatality rates, it is believed that as much as 120000 deaths may be attributed to cholera every year [1].

The patogenicity of the *Vibrio cholerae* strains belonging to serogroup O1 and O139 is due to the production of virulence factors such as cholera toxin (CT) and the toxin-coregulated pilus (TCP); CT is essential for full-blown cholera whereas TCP is crucial for the adherence of bacteria to the intestinal epithelium [2], [3]. The remaining serogroups, referred to as non-O1 and non-O139, which mostly lack CT and TCP, are more frequently isolated from aquatic environmental sources than from clinical samples; nevertheless, these strains have been reported to evoke fluid accumulation in the ligated rabbit ileal loop assay [4]–[6] and to cause human disease, such as sporadic outbreaks of watery diarrhoea and inflammatory enterocolitis, despite the absence of CT. Nontoxigenic O1 strains have also been isolated from cases of diarrhoea [7]–[9]. All these evidence suggested the possibility that other virulence factor(s) than CT might be crucial in the pathogenesis of *Vibrio cholerae*-induced diarrhoea, but their nature remains still unknown.

El Tor hemolysin (ETH), also known as *V. cholerae* cytolysin (VCC) consists of two major toxin groups, namely *V. cholerae* O1 (VCC1) and *V. cholerae* non-O1 (VCC2) and is produced by all strains. It is a water soluble toxin secreted as a 79 kDa inactive pro-hemolysin by *V. cholerae* [10], [11]. A proteolytic cleavage by different proteases, that remove a N-terminal segment [12], generates the mature toxin of 63 kDa. In cholesterol-and ceramides-rich membranes VCC forms heptameric channels [13], [14] with a moderate anion preference, responsible for vacuolization and eventual lysis of several cell types in culture [15]–[18]. Recently, the vacuolization associated to VCC intoxication has been demonstrated to be an autophagic response of the cells against the toxin [19]. VCC has been proposed as virulence factor causing diarrhoea [6], [20], although the ability of non-haemolytic strains of *V. cholerae* to cause fluid accumulation, suggests that it is not the sole etiologic factor involved [21].

On the basis of its channel property and considering that the majority of the diarrheagenic toxins affect chloride secretion in the intestine [22], we have investigated whether VCC was able to promote an efflux of chloride from intestinal epithelial cells either by using an intestinal cell line or by administrating the toxin on a human intestinal mucosal sheet.

Here we demonstrate for the first time that VCC is capable of inducing a chloride efflux from whole human intact intestinal epithelium and in virtue of such an activity we suggest that VCC might be the major diarrheagenic factor for the non producing cholera toxin strains, or contribute to cause diarrhoea when the toxin is present.

## Materials and Methods

### Reagents

**Figure 1:**
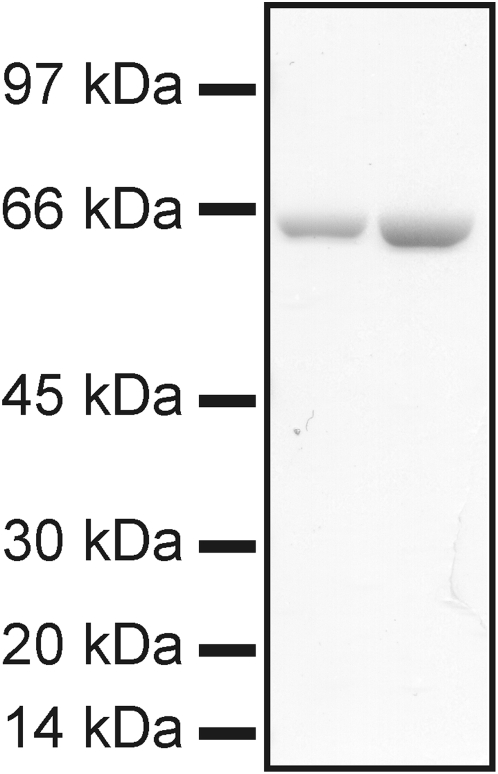
SDS-PAGE of VCC preparation. 0.5 and 1 µg (left and right lane respectively) of VCC, purified as described in *Materials and Methods*, were applied to SDS-PAGE and stained with Comassie-blue.

*Vibrio cholerae* cytolysin (VCC) was purified from culture supernatants of *V. cholerae* O1 El Tor 8731 [23]. The cleaved and active form of VCC was obtained by ethanol precipitation (final concentration, 40%), preparative isoelectric focusing in a sucrose density gradient, and hydroxyapatite chromatography [13], [24]. Figure 1 shows the purity of the VCC used for the experiments. Cell culture media and FBS were from Invitrogen/Gibco. MEQ was from Molecular Probes. Krebs buffer, Staphylococcus aureus α-Hemolysin, 4,4′-diisothiocyanatostilbene-2,2′-disulphonic acid (disodium salt) (DIDS), protamine, amiloride, papain, L-cysteine, FITC and Na-Isethionate were from Sigma. Caco-2 cells were from Istituto Zooprofilattico Sperimentale (Brescia, Italy).

### Caco-2 cells culture

Caco-2 cells, obtained from human colon adenocarcinoma, were grown in DMEM supplemented with 10% FBS. For chloride measurements cells were harvested the day before and seeded on 24 mm-glass coverslips (4×10^5^cells).

### Intracellular Cl^−^ measurements

Changes of intracellular Cl^−^ concentration were measured with the chloride indicator, 6-methoxy-N-ethylquinolinium iodide (MEQ) [25]. MEQ fluorescence is quenched by collision with halide ions, thus an increase in the fluorescent signal represents a decrease in the intracellular Cl^−^ concentration. Caco-2 cells, seeded on coverslips, were loaded with 50 µM diH-MEQ in solution #1 (NaCl 135 mM, KCl 4.7 mM, KH_2_PO_4_ 1.2 mM, MgSO_4_•7H_2_O 1 mM, CaCl_2_ 1.2 mM, HEPES 10 mM, glucose 5 mM, pyruvic acid-sodium salt 1 mM, pH 7.4) for 50 min before washing and visualizing on an Olympus inverted microscope. The incubation with VCC or PBS (as control) was carried out during the last 30 min of the dye loading. Cells were maintained in continuous perfusion with the chloride-containing buffer (solution #1): after 340 sec, solution 1 was substituted with a Cl^−^ free buffer (solution # 2) containing NaNO_3_ 140 mM, KNO_3_ 5 mM , gluconic acid 3 mM, MgSO_4_•7H_2_O 1 mM, glucose 5 mM, HEPES 20 mM, pH 7.4. After 500 sec, cells were re-exposed to solution 1 for 500 sec. For each experiment, fluorescence (excitation at 360 nm; emission at 420 nm) from microscopic fields, containing an average of 2–3 cells, was recorded (1 recording every 10 sec) and quantified.

To quantify changes in cellular fluorescence, the total quenchable signal of the dye was determined in each experiment, and expressed as the difference between the fluorescence signal when the cells were perfused with chloride-free buffer and the signal obtained after maximum quenching by perfusion with 150 mM potassium thiocyanate (KSCN). In the histogram representation the change in fluorescence from baseline (ΔF) was calculated by the equation ΔF = (1−F/F_b_)×100, where F_b_ is the basal fluorescence at time = 0 sec. Using this method, each experiment was able to serve as its own control [26].

### Experiments on human colon

Sigmoid colon tissues were obtained from patients undergoing subtotal colectomy for colon cancer. Before surgery, all patients had given their fully informed and written consent about the aims of the surgical intervention. Upon removal of the tissue and before inclusion in buffered formaline for routine pathology examination, a strip (about 5×1.5 cm) of full thickness colonic wall was isolated within the redundant healthy area surrounding the tumor and kept for electrophysiological studies. During dissection and experiments tissues were continuously bathed with Krebs bicarbonate/phosphate buffer, containing in mM: 107 NaCl, 4.5 KCl, 25 NaHCO_3_, 0.2 NaH_2_PO_4_, 1.8 Na_2_HPO_4_, 1.25 CaCl_2_, 1 MgCl_2_, and 12 D-glucose, continually gassed with O_2_/CO_2_ (95%/5%) to yield pH 7.2, and warmed up to 37°C. After removal of serosal and circular muscle layers, the stripped mucosae were mounted vertically as flat sheet between the two halves of Ussing chambers (Mussler Scientific Instruments, Aachen, Germany), having an exposed area of 1 cm^2^. Each half-chamber had a circular fluid canal of 3.0 ml total volume filled with Krebs solution that was constantly recirculated by means of gas bubble lift. Two pairs of Ag/AgCl electrodes were used to monitor either the transepithelial potential difference (V_T_, mV) and the tissue resistance (R_T_, Ω cm^2^) under open-circuit condition, or the short circuit current (I_SC_, µA/cm^2^) with the transepithelial potential clamped to zero at fixed intervals of 5 min. Offset of voltage electrodes pairs and fluid resistance were evaluated prior to the onset of each experiment and systematically subtracted. At fixed intervals of 1 min a transepithelial bipolar current pulse (I) of 1 µA amplitude and 200 msec duration was applied to the tissue and the R_T_ was automatically calculated from the change in open-circuit voltage (ΔV_T_) according to Ohm's law (R_T_ = ΔV_T_/I).

Experiments were conducted simultaneously on one to three specimen from the same tissue. Electrical parameters were measured in the computer-controlled chambers (software Clamp v. 2.14, Aachen, Germany) in the basal state (i.e. an equilibration time of 30 to 40 min), during incubation with VCC, α-Hemolysin or drugs added to the mucosal side and after removing the agonists. In some experiments Na-Isethionate was used to completely replace the NaCl when Cl^−^ was reduced from 116 to 9 mM in the luminal and mucosal bath. In order to avoid Ag/AgCl electrode offset change during Cl^−^ substitution, the tissues were incubated in low-Cl^−^ solution from the beginning of the experiment.

To enhance VCC interaction with mucosal colon surface, the layer of mucus was removed by incubation in a mucolytic solution containing papain (5 U/100 ml) plus L-cysteine (5 mM) [27], [28], for 25 min. The luminal mucolytic containing solution was then substituted with Krebs solution 5 min prior the addition of the toxin. The enzyme concentration and the exposure time were kept low to avoid damage to colon mucosa. In order to prevent cysteine formation and precipitation, the mixture was freshly prepared prior each experiment.

VCC, α-Hemolysin and drugs were added directly to the mucosal bath, while solution replacing after the mucolytic treatment or drug addition was obtained within about 40 sec by continuous gravity perfusion from a reservoir and suction of the mucosal bath excess by a vacuum pump.

### Paracellular permeability evaluation in intact intestinal epithelia

The mucosal-to-serosal flux of fluorescein isothiocyanate (FITC; MW: 376.3) in human colon specimen mounted in Ussing chamber was assessed as described by Mayol and colleagues [29]. Tissue were incubated in Krebs buffer and exposed to mucolytic solution as described above. After mucolytic cocktail removal, FITC was added in the mucosal bath (140 µM final concentration), with or without VCC (1 nM) or protamine (100 mg/l). Serosal buffer aliquots were collected at 0, 5, 15, 30 and 60 min after FITC addition and replaced with identical PBS amount. The fluorescent emission at 520 nm after excitation at 480 nm was measured twice for each aliquot with a fluorescence spectrophotometer equipped with microplate reader (Cary Eclipse, Varian Inc. Palo Alto, CA, USA). A calibration curve obtained with fluorescence intensity (expressed as arbitrary units of the optical density measured at 480 nm) *vs* FITC concentration was generated to calculate the FITC concentration in the serosal chamber. The apparent permeability coefficient (P_app_) was calculated using the equation P_app_ (cm/sec) = ΔC_s_×C_m_^−1^×V×A^−1^×T^−1^, where ΔC_s_ is the increase in FITC concentration in the serosal chamber during the interval T (sec), C_m_ is the FITC concentration in the mucosal chamber, V is the volume (ml) of either the mucosal or serosal compartment and A is the exposed surface of the colon specimen (cm^2^). Serosal (C_s_) and mucosal (C_m_) FITC concentrations were corrected respectively for dilution and flux.

### Statistical analyses

Data are expressed as means±S.E.M. Student's *t*-test was used for statistical analysis of differences between experimental groups. In experiments on human colon each data set represents independent measurements on separate specimen (n) obtained from stripped mucosal preparation (m), were one to three tissue specimen were obtained from the same colon. Student's *t*-test for paired data was performed between results obtained on separate specimen (n) in treated *vs* untreated condition. A *p*-value equal or below 0.05 was defined as a significant difference. Calculations were performed with the NCSS2007 software (Hintze J. Kaysville, UT, USA, www.NCSS.com).

## Results

### VCC induces chloride efflux from Caco-2 cells

**Figure 2:**
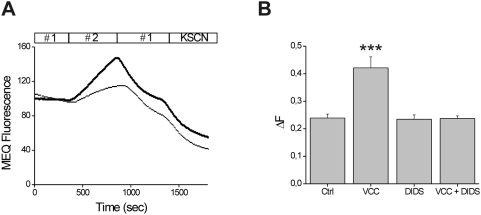
Chloride efflux induced by VCC in Caco-2 cells. A) Caco-2 cells, loaded with MEQ were incubated with (thick line) or without (thin line) 500 pM VCC. Fluorescence was estimated upon sequential buffer substitution with Cl^−^- containing medium (# 1) followed by Cl^−^-free medium (# 2). Substitution of solution (#1) by solution (#2) led to an increase of MEQ fluorescence (dequenching). Subsequent substitution by solution #1 induced an influx of Cl^−^ ions, thus quenching MEQ fluorescence. Values are normalized with respect to the base line. B) Effect of 100 µM DIDS on VCC-induced chloride efflux. ΔF has been calculated as reported in the *Material and Methods* section. Each histogram represents an average of thirteen independent experiments with a recording of 2–3 cells in each assay. Significance, determined by Student's *t* test, was compared to non-treated cells (Ctrl); ***, p<0.001.

Diarrheagenic toxins affect Cl^−^ secretion in the intestine. Based on its channel properties, as discussed above, we first evaluated whether VCC was able to promote a chloride efflux in the Caco-2 intestinal cell line. Chloride movement was monitored using the chloride-sensitive fluorescent dye MEQ, whose increase in fluorescence indicates chloride efflux from the cytoplasm. Cells loaded with diH-MEQ were incubated with PBS (control) or with 500 pM VCC for 30 minutes before each experiment. After a basal record of fluorescence in Cl^−^-containing HEPES buffer, cells were perfused with a chloride-free buffer to induce a chloride efflux from the cytoplasm and subsequently exposed to HEPES buffer to allow the chloride readmission. As shown in Fig. 2A this manoeuvre induced in the control cells an increase in fluorescence followed by a decrease, suggesting the presence of functional chloride channels on the plasma membrane. In cells treated with VCC the extent of increased fluorescence was significantly higher suggesting an increase in chloride permeability. This effect could be mediated by the activation of endogenous channels by the toxin or by the toxin itself. To discriminate between these two alternative possibilities the effect of the chloride channel inhibitor DIDS, an efficient blocker of the VCC-formed channels [18], was evaluated on control and VCC-treated cells. As shown in Fig. 2B, incubation with 100 µM DIDS did not affect the fluorescence signal of control cells, demonstrating that the endogenous channels were not blocked by DIDS at the concentration used. In contrast, DIDS completely abrogated the enhanced fluorescence signal of toxin-treated cells. These data strongly suggest that VCC forms chloride channels in the plasma membrane of the cells.

### VCC determines short circuit current (Isc) increase and transepithelial resistance (R_T_) decrease in the whole intact intestinal epithelium

In subsequent experiments, we assessed whether VCC acts as Cl^−^ channel also in a human model of the natural anatomical site of the bacterial colonization. *V. cholerae* infection affects both small and large bowel [30], however, the possibility to obtain human jejunum rather than colon specimen is scanty, whilst large intestine specimens are more common. Accordingly, we used human healthy sigmoid colon mucosal sheets, obtained from 47 patients (28 male and 19 female; ages between 32 and 84) undergoing subtotal colectomy for colon cancer. A total of 62 specimens (n) from 37 tissues (m), mounted in Ussing chambers at 37°C and bathed in oxygenated Krebs solution, exhibited a basal V_T_ value of −7.28±(S.E.M.) 0.49 mV, lumen negative; R_T_ was 149.24±5.22 Ω cm^2^ and I_SC_ was 37.16±1.57 µA/cm^2^. Topical exposure to VCC (0.2, 0.5, 1 nM) did not elicit significant changes within 90 min (n = 8; m = 6), data not shown. The inefficacy of VCC in these experiments could be related to the presence of the protective mucus layer coating the gut lumen [31], [32], which is however penetrated by vibrions. Therefore, in order to improve VCC interaction with the apical membranes of colon enterocytes, the luminal mucus layer was removed by enzymatic exposure (as explained in the *Material and Methods* section).

**Figure 3:**
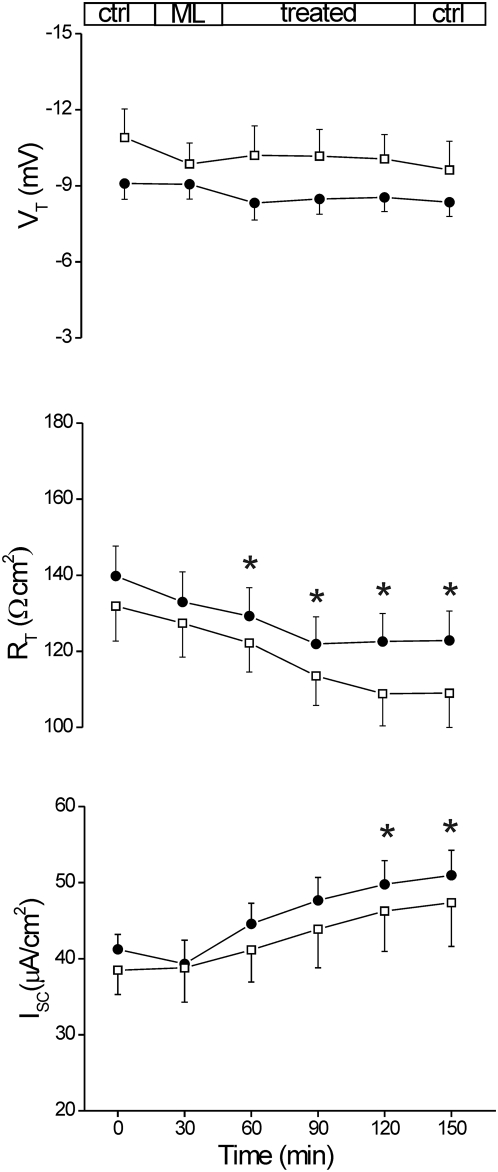
Effect of mucosal exposure to VCC. Human sigmoid colon specimens mounted in Ussing chamber were sequentially monitored for transepithelial potential difference (V_T_, mV) and tissue resistance (R_T_, Ω cm^2^) changes under open-circuit condition, and for short circuit current (I_SC_, µA/cm^2^) with voltage clamped to zero. Electrical parameters were measured in the basal state (Ctrl) and during incubation (Treated) with VCC 1 nM (filled circles: n = 10; m = 7) or 0.5 nM (empty squares: n = 5; m = 2). All tissues were treated with mucolytic solution (ML) containing papain (5 U/100 ml) plus L-cysteine (5 mM) prior exposure to VCC. Each data point represents the average±S.E.M. of measurements in n specimen from m mucosae. Significance was determined by Student's *t* test for paired data of treated *vs* control (time = 0); *, p<0.05.

**Figure 4:**
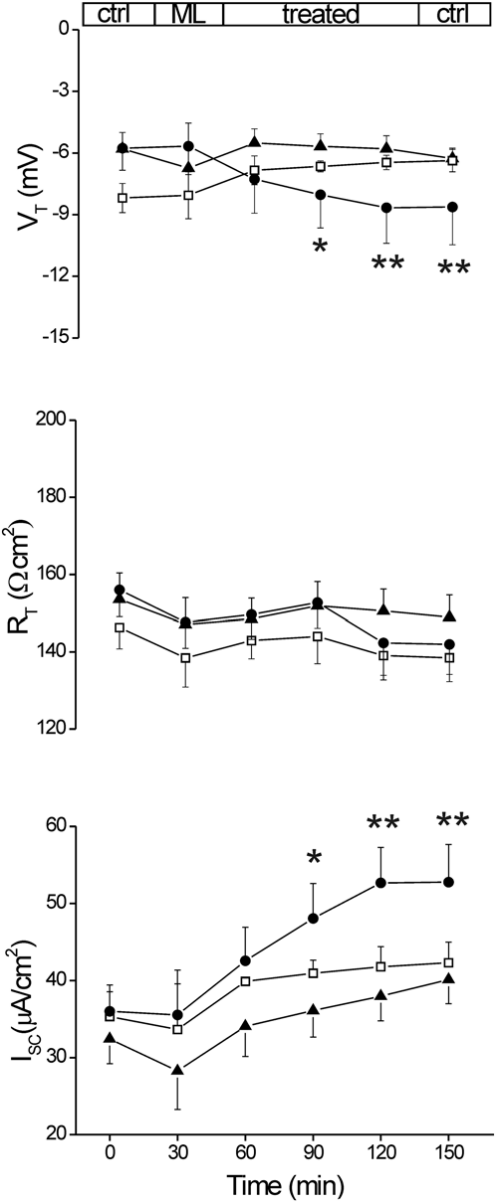
Effect of mucosal exposure to Staphylococcus aureus α-hemolysin. Human sigmoid colon specimens in the basal state (Ctrl) and during mucosal incubation (Treated) with α-hemolysin 60 nM (filled triangles: n = 5; m = 4), 90 nM (empty squares: n = 3; m = 3) and 120 nM (filled circles: n = 6; m = 6). Electrical parameters were measured as reported in legend of Fig. 3. Each data point represents the average±S.E.M. of measurements in n specimen from m mucosae. Significance was determined by Student's *t* test for paired data of treated *vs* control (time = 0); *, p<0.05; **, p<0.02.

Following removal of the enzyme cocktail, exposure to 1 nM VCC (n = 10; m = 7) within 90 min increased significantly I_SC_ by 20.7% (p<0.05 *vs* control) and reduced R_T_ by 12.3% (p<0.05) while V_T_ decreased by about 7% but not significantly, as reported in Fig. 3. Following the same protocol, the exposure to 0.5 nM VCC (n = 5; m = 2) elicited similar responses but with smaller significance (Fig. 3). R_T_ reduction and I_SC_ increase reflect alteration of the epithelial permeability and enhancement of the ion flux which might be related to VCC-dependent channel formation. In order to test the sensitivity of the human colon epithelial model to pore-forming bacterial toxins, a series of comparative experiments was performed using α-Hemolysin of *Staphylococcus aureus* [33], [34]. Following similar protocol for mucus layer removal by enzymatic treatment, exposure to 120 nM α-Hemolysin within 90 min increased significantly I_SC_ by 46.3% (p<0.02 *vs* control), while V_T_ became more negative by 49.9% (p<0.02 *vs* control) and R_T_ was reduced by 8.7%, albeit not significantly (Fig. 4). Similar, although minor, responses were observed with 60 and 90 nM α-Hemolysin.. Alteration of the epithelial parameters points to a correlation with toxin-dependent channel formation.

### VCC did not affect paracellular permeability

**Figure 5:**
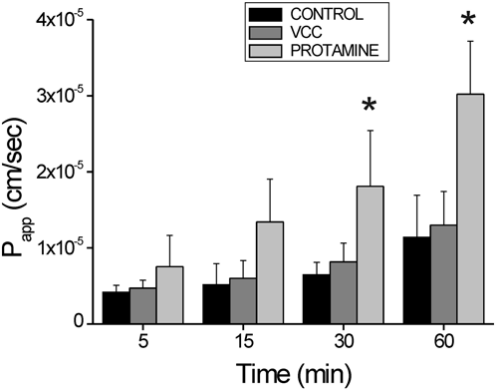
Effect of VCC on paracellular permeability. Paracellular permeability of human colon specimen (0.8 cm^2^) mounted in Ussing chamber to mucosal-to-serosal flux of fluorescein isothiocyanate (FITC 140 µM) was measured in the presence or absence of mucosal VCC (1 nM) or protamine (100 mg/ml). Data are expressed as apparent permeability (P_app_) calculated as reported in the *Material and Methods*. Each data point represents the average of 4 independent experiments. T-test for paired data was performed *vs* control (PBS exposed mucosa): * p<0.05.

It is widely accepted that trans-epithelial resistance is an accurate marker of epithelial viability and correlates with selective mucosal permeability [35], [36]. In order to investigate whether a paracellular ion flux could contribute to the VCC-induced R_T_ reduction, the permeability of colon mucosa was assessed by mucosal-to-serosal FITC flux [29]. Fig. 5 shows that FITC apparent permeability after mucosal exposure to VCC (1 nM) for 60 min was 1.3×10^−5^±4.4×10^−6^ cm/sec (n = 4), a value not significantly different from 1.1×10^−5^±5.5×10^−6^ cm/sec (n = 4), observed in the presence of VCC vehicle (PBS). Dissimilarly, mucosal exposure to the polyamine protamine increased significantly epithelial permeability up to 3.2×10^−5^±6.9×10^−6^ cm/sec (n = 4; p<0.05 *vs* control), as observed in other substrates [37].

### VCC-induced effects on intestinal epithelium depend on chloride channels

**Figure 6:**
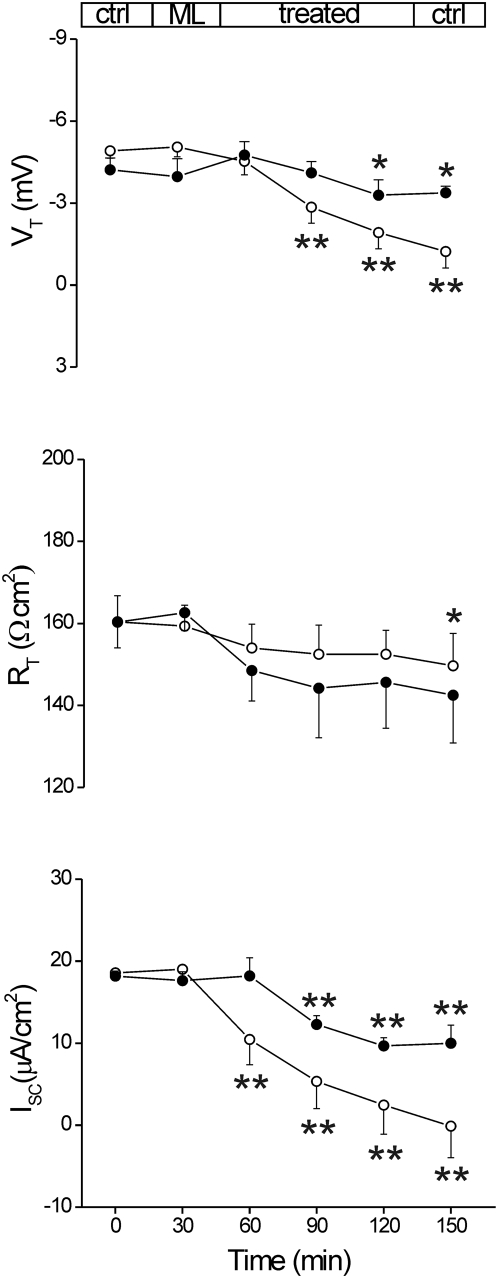
Effect of VCC during bilateral incubation in low-Cl^−^ solution. The colon specimens mounted in Ussing chamber were incubated in low-Cl^−^ (9 mM) solution, from the beginning to the end of the experiment, in the presence or in the absence of mucosal VCC 1 nM. Electrical parameters were measured as reported in legend of Fig. 2. Filled circles refer to toxin-exposed tissues (n = 3; m = 3), while empty circles refer to non-treated tissues (n = 4; m = 3). Significance was determined by Student's *t* test for paired data of control (time = 0) and treated; *, p<0.05;**, p<0.02.

Correlation between the observed effects and putative VCC chloride channels was tested by lowering Cl^−^ in the mucosal and serosal solution from 116 mM to 9 mM (NaCl replaced with Na-Isethionate). If chloride channels are functional in the cell plasma membrane this manoeuvre should lead to diminution of the VCC effect on I_SC_ but not on R_T_ since Cl^−^ reduction would affect the ion transport but not channel formation. Fig. 6 shows that in 4 specimen from 3 tissues bilateral incubation with low-Cl^−^ reduced V_T_ by 60.9% (p<0.02) and I_SC_ by 86.8% (p<0.02) up to current polarity inversion. R_T_ was reduced only by 4.9%. These changes could be interpreted as a consequence of the reduced Cl^−^ secretion from the crypts [30]. Exposure to 1 nM VCC during bilateral chloride diminution (n = 3; m = 3), reduced I_SC_ and V_T_ by 46.8% (p<0.02) and 22.0% (p<0.05) respectively, while R_T_ was reduced by 9.1%. The variations reflect the increase of the epithelial permeability related to chloride channel formation and its influence on ion transport in both the crypt and surface epithelial cells.

### VCC-induced effects are independent on Na^+^ transport

**Figure 7:**
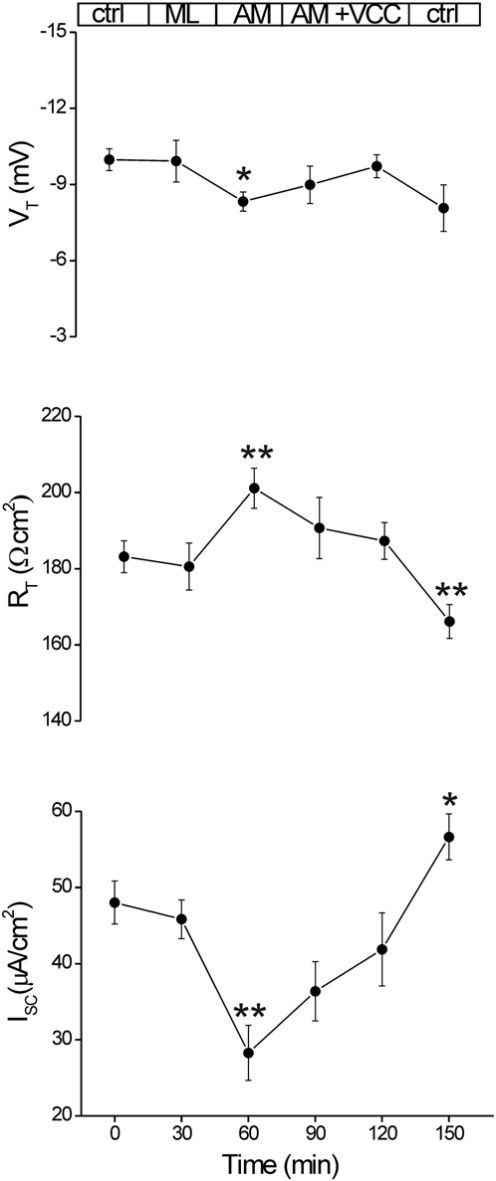
Effect of amiloride on VCC-induced Isc. Effect of VCC (1 nM) on Na^+^ conductance was tested on amiloride (0.1 mM) pretreated human sigmoid colon (n = 4; m = 3). Electrical parameters were measured in the basal state (Ctrl), followed by incubation with mucosal amiloride (30 min), successive addition of VCC (60 min) and after removing the agonists (Ctrl). Significance was determined by Student's *t* test for paired data of control (time = 0) and treated; *, p<0.05;**, p<0.02.

Since VCC-induced current increase could depend either on the Cl^−^ secretion or on the electrogenic Na^+^ absorption, the potential relationship between the VCC-induced current increase and Na^+^ transport was tested using the Na^+^ channel blocker amiloride [38], [39]. Fig. 7 shows that in 4 specimen from 3 tissues amiloride (0.1 mM) significantly reduced the I_SC_ and the V_T_ by 41.1% (p<0.02) and 16.5% (p<0.05) respectively, while R_T_ increased by 9.8% (p<0.02). Subsequent exposure to 1 nM VCC in 1 hour increased I_SC_ and V_T_ toward control levels up to 28% and 14% respectively, while R_T_ was reduced by 7.5%. Replacing the mucosal bath with Krebs solution removed the effects of amiloride restoring the I_SC_ almost to the control value, while dropped the R_T_ to about 9.3% below control value. The latter effect was probably due to the formation of VCC-induced channels.

### VCC contributes to the endogenous chloride movement through the intestinal epithelium

**Figure 8:**
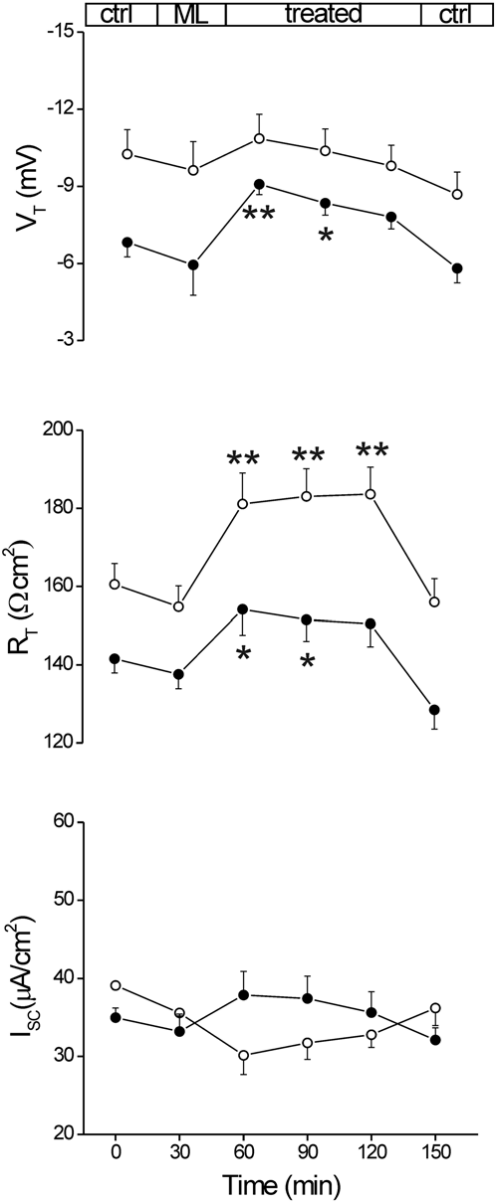
Effect of DIDS on VCC-induced Isc. Effect of VCC on human sigmoid colon Cl^−^ conductance was tested by mucosal exposure to DIDS (1 mM) in the presence (n = 7; m = 4) or in the absence (n = 7; m = 5) of VCC 1 nM. Filled circles refer to toxin-exposed tissues while empty circles refer to non-treated tissues. Electrical parameters were measured in the basal state (Ctrl), during incubation with DIDS or DIDS plus VCC added to the mucosal side (Treated) and after removing the agonists (Ctrl). Significance was determined by Student's *t* test for paired data of control (time = 0) and treated; *, p<0.05;**, p<0.02.

In order to better analyze the Cl^−^ ion movement that occurred through the VCC-formed pores we used the chloride channel inhibitor DIDS, on both control tissue and toxin-treated colon, at a concentration able to block chloride transport in apical membrane vesicles from human distal colon [40], and in the whole rat colon [41]. As reported in Fig. 8, DIDS (1 mM), after 90 min of incubation, increased significantly R_T_ by 14.4% (p<0.02; n = 7; m = 5). I_SC_ and V_T_ were both altered transiently in the first 30 min: I_SC_ decreased with a maximal reduction of 22.9%, while V_T_ increased by 5.9%, albeit both changes were not significant. R_T_ increase is suitable with apical endogenous anionic channel blockage, while transient V_T_ increase and I_SC_ decrease could probably be related to the progressive reduction of both the basolateral Na^+^-K^+^-2Cl^−^ cotransport activity and the K^+^ recirculation through the basolateral K^+^ channels following blockage of apical Cl^−^ exit [30], [41], [42]. Exposure to 1 nM VCC at the same time of DIDS transiently increased V_T_ within 30 min by 33.1% (p<0.02) and I_SC_ by 8.2% (n = 7; m = 4). The increase could be probably interpreted as progressive appearance and blockage of VCC-formed channels. The VCC-induced R_T_ drop was partially inhibited by DIDS. An analysis comparing the three sets of experiments (exposure to VCC, DIDS and VCC+DIDS) and converting the epithelial resistance into conductance indicates that DIDS inhibits by about 70% the toxin anion channels. Taken together, these findings suggest that VCC-formed channels can support additional apical chloride efflux from human colon tissue samples.

## Discussion

The mechanism involved in the pathogenesis of human enteritis consequent to infections sustained by non-toxigenic *Vibrio cholerae* strains remains still unclear [9], [43], [44] and no single virulence factor has yet been identified to explain their enterotoxicity [45]. VCC, the hemolysin produced by virtually all *Vibrio cholerae* strains, has been proposed as a possible candidate: Hichinose and colleagues, first reported the enterotoxicity of El Tor-like hemolysin from non-O1 *V. cholerae* in 1987 [20], a finding that was later confirmed [6], [46]. However, although collectively these data suggest a crucial role for VCC as an enterotoxic virulence factor, a clear-cut demonstration is still lacking.

Considering that one of the mechanisms of toxin-induced diarrhoea depends on direct effects on ion transport in intestinal epithelial cells [22], we examined the effects of purified VCC on human healthy sigmoid colon mucosal sheets. The sensitivity of the human colon epithelial model to pore-forming bacterial toxins was also assessed by comparative experiments using α-Hemolysin of *Staphylococcus aureus*.

The exposure to the VCC significantly reduced R_T_ up to 12.3% and increased I_SC_ up to 20.7% within 90 min.

The R_T_ decrease could be the result of change either in the epithelial cell membrane conductance or in the paracellular pathway. Evaluation of the mucosal-to-serosal flux of fluorescein isothiocyanate demonstrated that paracellular pathway is not affected by the VCC, thus the R_T_ decrease could be related to modification of the transcellular resistance and represents the major evidence that VCC forms pores. The analysis of time course for R_T_ and I_SC_ changes upon VCC exposure suggests that, for the concentrations used in our experiments, the channel formation is a gradual process that reaches its maximum within one hour.

In the absence of VCC, and with a null mucosal *vs* serosal ionic gradient, the polarization of the whole epithelium depends either on the Cl^−^ secretion or on the electrogenic Na^+^ absorption. In the distal colon the Cl^−^ secretion occurs mainly through the cystic fibrosis transmembrane conductance regulator channels (CFTR), which is expressed throughout the entire colonic epithelium but dominates in the lower part of the crypts [30]. The Na^+^ transport relies mainly on amiloride-sensitive Na^+^ channels (ENaC) expressed in the surface epithelium and upper crypts [30]. On the basis of these considerations, the I_SC_ increase consequent to the VCC exposure could be compatible with the enhancement of either the Cl^−^ secretion or the Na^+^ absorption.

If, by hypothesis, VCC forms cation channels, as suggested in a previous report [24], we would expect a rise of the I_SC_ associated to an increased electrogenic Na^+^ flux. Our experiments with amiloride, a typical inhibitor of the Na^+^ conductance [39], showed that the VCC-dependent I_SC_ increase, as well as the R_T_ decrease, are not affected by the loop diuretic, excluding the Na^+^ flux involvement in the VCC-induced effects.

In order to confirm that the I_SC_ increase, observed upon VCC exposure, was due to the Cl^−^ ion passage through VCC-formed channels, we evaluated the effects of the toxin after reducing Cl^−^ concentration in the incubation milieu. Mucosal VCC addition elicited a large drop of the R_T_ compatible with channel formation in the apical membrane and responsible for a conductance increment which is virtually independent from the ion concentration. In contrast, the I_SC_, which remained almost constant during the first 30 minutes, probably because of progressive VCC-channel formation, subsequently started to decrease reaching 50% of the initial value. This demonstrated that the I_SC_ increment induced by the toxin (Fig. 3) is principally related to the Cl^−^ movement. Treatment with the chloride channel inhibitor DIDS also confirmed that the ion movement occurs through VCC-formed pores.

Altogether these results represent the first *in vivo* evidence that VCC could be a diarrhogenic factor. Indeed, abnormal secretion of Cl^−^ across the intestinal apical membrane would increase the outward movement of Na^+^ and water, leading to the accumulation of fluid in the gastro-intestinal tract.
